# A Comparison of Emergency Medical Service (EMS) Utilization, Dispatches for Respiratory Complaints, and Non-transports Before, During, and After the COVID-19 Pandemic

**DOI:** 10.7759/cureus.113540

**Published:** 2026-07-28

**Authors:** Sarah Awad-Sichel, Patrick Matthews, Keshab Subedi, Richard Baker, Jason Nomura, Robert Rosenbaum

**Affiliations:** 1 Emergency Medicine, Christiana Care Health System, Newark, USA; 2 Emergency Medical Services, Delaware Office of Emergency Medical Services, Smyrna, USA; 3 Institute for Research on Equity and Community Health (iREACH), Christiana Care Health System, Newark, USA; 4 Emergency Medicine Research, Christiana Care Health System, Newark, USA

**Keywords:** covid-19, emergency medical services, ems, pandemic, utilization

## Abstract

Introduction

COVID-19 caused massive healthcare system disruptions. The pandemic’s impact on Emergency Medical Service (EMS) utilization varied regionally and is still not fully characterized. Some data show decreased overall EMS utilization, changes in utilization for respiratory complaints, and increases in non-transports during the pandemic. Limited data are available on the changes in EMS utilization post-pandemic. This study analyzes changes in EMS utilization by comparing the pre-pandemic, pandemic, and post-pandemic periods.

Materials and methods

This is a retrospective analysis of the Delaware EMS database from January 2019 to July 2022. Time periods included “pre-pandemic” (January 1, 2019, to March 24, 2020), “pandemic” (March 24, 2020, to May 30, 2020), and “post-pandemic” (May 31, 2020, to July 1, 2022). The pandemic period corresponds to the statewide "stay-at-home" order, representing the period with the greatest restrictions. Call volume was based on basic life support responses. Daily call volume, percentage of respiratory dispatches, and percentage of non-transports from pre-pandemic, pandemic, and post-pandemic periods were compared while controlling for seasonality.

Results

There were 155,781 pre-pandemic calls, 19,584 pandemic calls, and 272,048 post-pandemic calls analyzed. Average daily pandemic EMS utilization decreased by 64.66 dispatches compared to pre-pandemic (95% CI: 55.72, 73.44; p < 0.001). Utilization pre-pandemic was 1.23 times higher than the pandemic period. Pre- and post-pandemic utilization did not differ significantly, although post-pandemic call volume showed a numerical increase in daily post-pandemic call volume by 7.32 (95% CI: 3.51-11.06; p = 0.09). The average pandemic daily activations for respiratory complaints as a percentage of total activations significantly increased by 1.41 (95% CI: 0.91, 1.90) compared to pre-pandemic (p < 0.001). Compared to pre-pandemic, the rates of non-transports increased significantly by 4.29 (95% CI: 3.62, 4.96; p < 0.001) during the pandemic and 1.99 (95% CI: 1.71, 2.28; p < 0.001) post-pandemic.

Conclusions

Analysis of overall EMS utilization in Delaware during the COVID-19 pandemic showed a statistically significant drop in EMS utilization during the pandemic and a nonsignificant increase in call volume post-pandemic when compared to pre-pandemic. The proportion of calls for respiratory complaints increased during the pandemic. Rates of patient non-transports increased during the pandemic and remained elevated in the post-pandemic period. Lifestyle changes, perceptions of danger, and/or the impact of governmental lockdowns may have contributed to these effects.

## Introduction

The COVID-19 global pandemic, caused by the viral agent SARS-CoV-2, has had massive impacts on the United States (US) healthcare system, including prehospital medicine. Emergency Medical Services (EMS) utilization in the US is still not fully understood, as the impact of the pandemic varied across regions.

Studies from the Midwest, Western Pennsylvania, Massachusetts, and Northern California found an initial decrease in call volume at the onset of the pandemic when stay-at-home orders were introduced as compared to call data from prior year(s) [1-4] Conversely, services in New York City saw an increase in call volume during the first month of the pandemic, followed by a decrease in calls during the second month when compared to the previous year [5].

Regarding EMS utilization by severity of conditions, the data broadly indicated a higher severity or acuity via an increase in calls requiring transport [1], an increase in high-acuity call types [5], and an increase in abnormal vital signs [2]. A study from Massachusetts found a decrease in calls for cardiac emergencies and strokes but an increase in calls for cardiac arrest and respiratory complaints [3]. Similarly, a study from Israel also noted an overall decrease in volume, an increase in cardiac arrest and infectious disease calls, and a rise in patient refusals compared with 2019 [6].

A larger study of national EMS data using the National Emergency Medical Services Information System found that ambulance calls resulting in transport, EMS activations, and volume of calls for pulmonary and general complaints increased as the incidence of COVID-19 increased at a county level. This study did not find an increase in the volume of cardiac arrest calls in areas with higher incidences of COVID-19 cases or deaths [7].

A recently published study from Strum et al. in Canada looked at paramedic call volume and transports before, during, and after the COVID-19 pandemic from 2016 to 2023. This study defined the pandemic period as February 2020 until April 2021, a cutoff aligned with decreased rates of COVID-19 cases in the area and a reduction in COVID-19-related critical illness. While the study found a decrease in 911 calls and transports during the pandemic consistent with most studies above, it notably found a substantial increase in both 911 calls and emergency department (ED) transports during the post-pandemic period. Despite the overall increase in transports, a decrease in the proportion of patients transported to the ED between pre- and post-pandemic periods (75.4% to 67.2%) was also significant [8]. At the time of publication, this is the only paper that also evaluates pre- and post-pandemic EMS call volume and transports.

The state of Delaware initiated a statewide “stay-at-home” order on March 24, 2020, shortly after the first reported COVID-19 case in the state [9]. Hospital systems were strained with increased volume and mortality related to the pandemic; however, EMS utilization in the state during and after this time is not yet fully understood. This study aimed to analyze changes in EMS utilization before, during, and after the pandemic by examining total EMS call volume, the percentage of respiratory-related EMS calls, and the percentage of non-transport calls during each period.

This work was previously presented as a poster abstract at the National Association of EMS Physicians 2025 Annual Meeting on January 9, 2025.

## Materials and methods

Study design

In a retrospective analysis, de-identified EMS call data were extracted from Delaware EMS call data for EMS activations from January 2019 to July 2022. This data was divided into three separate time periods: (i) “pre-pandemic” (1 January 2019 to 23 March 2020); (ii) “pandemic” (24 March 2020 to 30 May 2020); and (iii) “post-pandemic” (31 May 2020 to 1 July 2022). The pandemic period was defined based on the duration of Delaware’s “stay-at-home” order. While additional restrictions were sporadically in place for a total of a calendar year, the initial lockdown was the most restrictive. The most restrictive portion of the pandemic was used in this study as the “pandemic” phase to gain insight into the patterns of EMS responses during the most heightened sense of pandemic awareness by the general population. Inclusion criteria were all basic life support (BLS) calls throughout the pre-pandemic, pandemic, and post-pandemic time periods described above. Only BLS calls were included in the analysis, as those encompassed all EMS calls and transports. Delaware BLS units respond to every EMS activation and serve as the transporting unit for all EMS patients. Advanced life support (ALS) responses were not included, as ALS may or may not have responded to the incident. An ALS response would be determined by the EMS dispatcher using the Medical Priority Dispatch System™ or by BLS request after patient assessment. No BLS calls during this time were excluded from analyses. This protocol was reviewed and approved by the Christiana Care Institutional Review Board FWA00006557 (approval number: 43077).

Study setting and population

Delaware utilizes a two-tiered EMS system with BLS responding to every call. In contrast, county-based ALS responds in separate non-transport units dispatched in conjunction by call severity or complaint. A total of 72 prehospital BLS agencies exist throughout Delaware. Delaware is unique in its small size, ranking 49th in the US by area, with only three counties and 1,948.5 square miles of urban, suburban, and rural areas. Delaware is a state within the US and had a total population of approximately 990,000 during the study period, as per the 2020 Decennial Census. All three counties in Delaware utilize the same system for 911 dispatch, and all ALS and BLS EMS units utilize the same state-wide electronic prehospital patient care report (PCR) software.

Outcome measures

Three primary outcomes were examined: call volume, average percentage of daily respiratory dispatches, and average percentage of daily non-transport calls. Percentage of respiratory dispatches and patient non-transports were used to account for changes in overall daily call volume between time periods. The pre-pandemic period was compared with the pandemic and post-pandemic time periods for each outcome. Data were adjusted by month to control for potential seasonality.

Respiratory pathology was determined as the documented call complaint listed at the time of dispatch. Calls labeled as “breathing problems” at the time of dispatch were classified as the respiratory category and chosen to be most inclusive of all respiratory complaints. Non-transports included all calls listed as refusal, no patient found, public assist, or canceled when extracted from the PCR.

Data analysis

Baseline patient characteristics, including age, gender, and race, were summarized using mean (SD), count, and percent for pre-pandemic, pandemic, and post-pandemic time periods. No duplicate records were found as there was only one BLS transport per patient encounter, regardless of ALS response. Any missing demographic data was not included for the patient encounter, and only documented data was reported. Trends in daily EMS call volume, proportion of EMS calls with breathing complaints, and rates of patient transport versus non-transports were visualized using line plots. Next, the effect of the pandemic on daily EMS call volume, the daily percentage of respiratory-related dispatches, and the daily percentage of non-transports was evaluated using linear models. For each outcome, linear regression models were fit with each study period as the primary independent variable of interest. The pre-pandemic period served as the reference category for all comparisons. “Month” was included as a categorical covariate (12 levels: January through December, with January as reference) to control for seasonal variation in EMS utilization patterns that occur independent of the pandemic. Statistical significance was set at p < 0.05. All the analysis and data visualization were done in R version 4.4.1. (R Foundation for Statistical Computing, Vienna, Austria).

## Results

The study population included 447,413 BLS calls (overall mean (SD) age, 56.9 (23.2) years) in the pre-pandemic, pandemic, and post-pandemic periods. Patient characteristics were similar among the three periods. Race data were available for 60.2% of pre-pandemic patients, 62.6% of pandemic patients, and 55.1% of post-pandemic patients where race was documented (Table 1).

**Table 1:** Demographic characteristics of the patient populations across the pre-pandemic, pandemic, and post-pandemic study periods Values are presented as counts and percentages of documented cases, with mean age shown for each period. SD: standard deviation

Time	Total patients	Mean age (SD)	Women (n, %)	Men (n, %)	Race documented (n, %)	White (n, %)	Black (n, %)	Hispanic (n, %)	Asian (n, %)	Other/not specified (n, %)
Pre-pandemic	155,781	56.76 (23.59)	82,698 (53.1%)	73,083 (46.9%)	93,780 (60.2%)	57,206 (61.0%)	27,418 (29.2%)	3,510 (3.7%)	478 (0.51%)	5,168 (5.5%)
Pandemic	19,584	57.30 (22.50)	9,789 (50.0%)	9,795 (50.0%)	12,260 (62.6%)	7,258 (59.2%)	3,715 (30.3%)	539 (4.4%)	59 (0.48%)	689 (5.6%)
Post-pandemic	272,048	55.90 (22.90)	139,761 (51.4%)	132,287 (48.6%)	149,898 (55.1%)	89,639 (59.8%)	45,182 (30.1%)	6,499 (4.3%)	809 (0.54%)	7,769 (5.2%)

**Figure 1:**
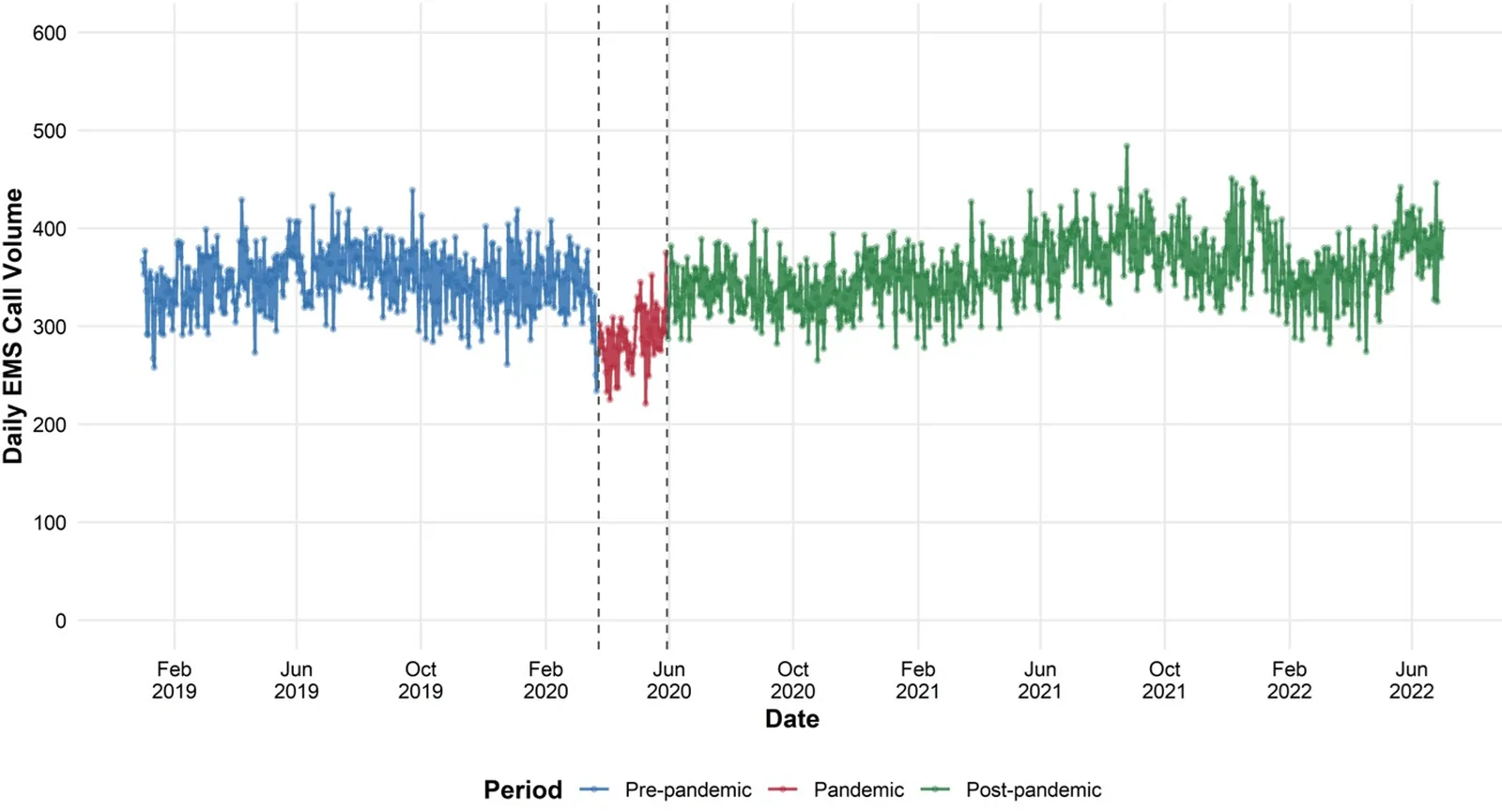
Average daily EMS utilization by call volume during the pre-pandemic, pandemic, and post-pandemic periods EMS: Emergency Medical Service

There was a significant decrease in EMS utilization from the pre-pandemic to the pandemic period (p < 0.001). The average daily EMS utilization during the pandemic period decreased by 64.66 dispatches (95% CI: 55.72, 73.44). The EMS utilization during the pre-pandemic period was 1.23 times higher than that during the pandemic period. EMS utilization during the post-pandemic period did not differ significantly from the EMS utilization during the pre-pandemic period (p = 0.09); however, the daily volume of EMS utilization showed an upward trend toward the end of the pandemic period and reached a higher level during the post-pandemic period compared to the pre-pandemic period. Compared to the pre-pandemic period, the average daily EMS utilization during the post-pandemic period increased by 7.32 (95% CI: 3.51, 11.06), although this difference was not statistically significant (Table 2, Figure 1).

**Table 2:** Total mean daily EMS call volume and breathing problem-related EMS call volume across the pre-pandemic, pandemic, and post-pandemic study periods Values are presented as the total daily volume of EMS calls, with 95% CIs shown for each group. EMS: Emergency Medical Service, CI: confidence interval

Period	Total mean daily EMS volume (95% CI)	Breathing problem mean daily EMS volume (95% CI)
Pre-pandemic	349.30 (346.29, 352.31)	31.56 (30.89, 32.23)
Pandemic	284.64 (276.24, 293.05)	29.67 (27.79, 31.55)
Post-pandemic	356.62 (354.34, 358.90)	33.49 (32.98, 34.00)

**Figure 2:**
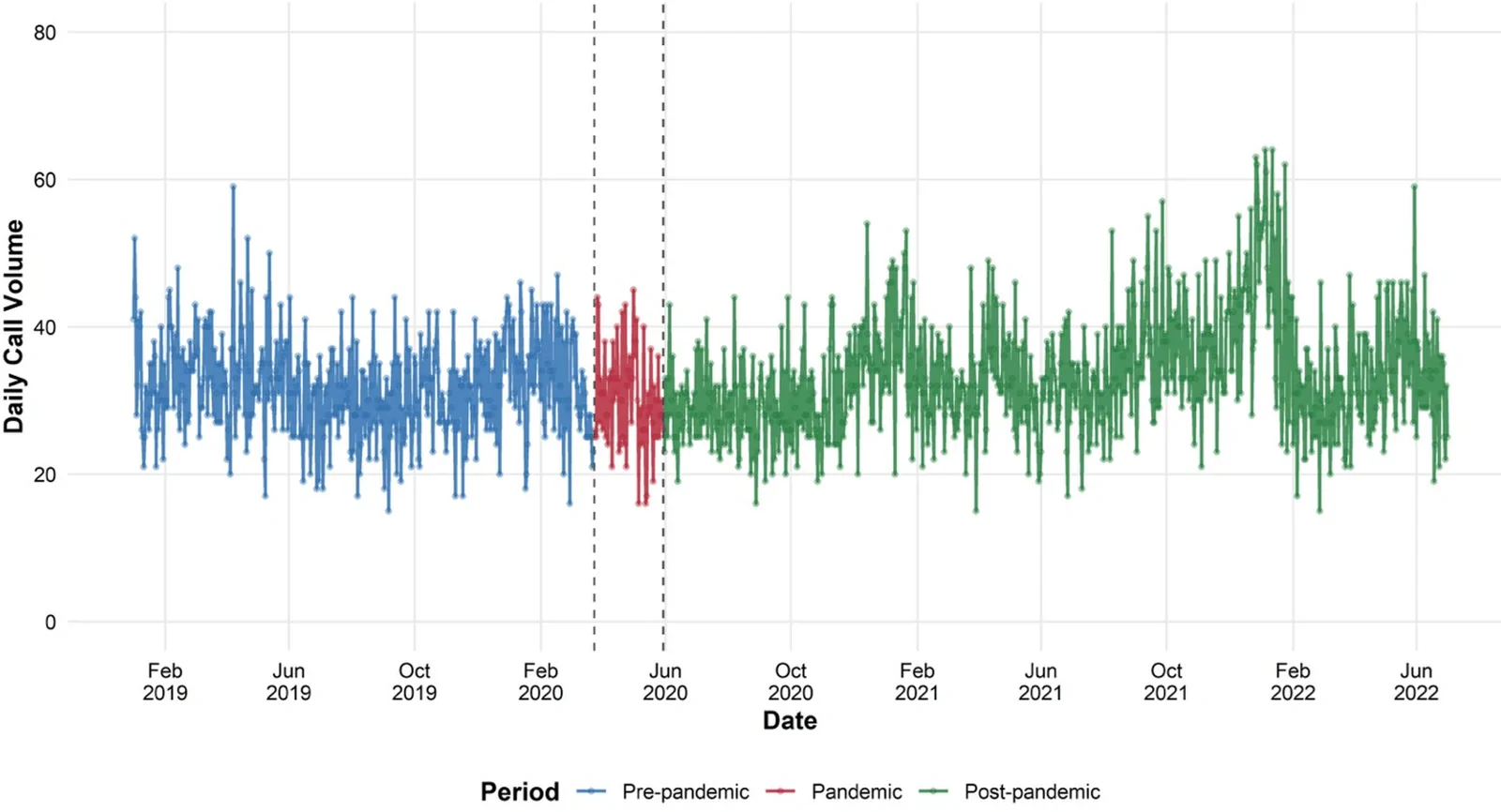
Average daily EMS utilization by call volume for respiratory complaints during the pre-pandemic, pandemic, and post-pandemic periods EMS: Emergency Medical Service

**Figure 3:**
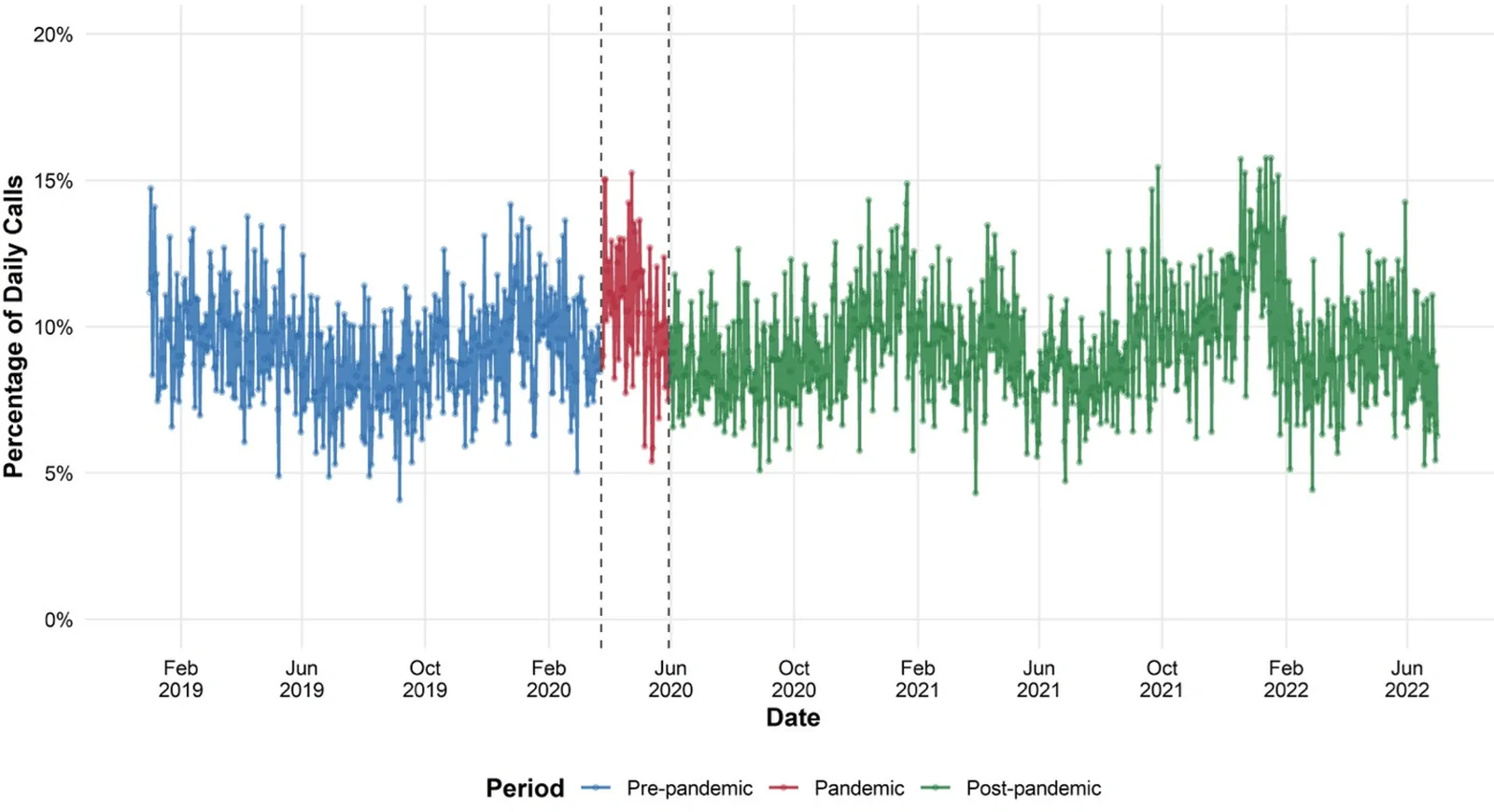
Average daily EMS utilization by percentage of call volume for respiratory complaints during the pre-pandemic, pandemic, and post-pandemic periods EMS: Emergency Medical Service

Average daily respiratory activations as a percentage of total activations with 95% CIs were 9.05% pre-pandemic (8.89%, 9.22%), 10.46% pandemic (9.99%, 10.92%), and 9.37% post-pandemic (9.25%, 9.50%). The percentage of daily EMS utilization for respiratory complaints significantly increased by 1.41 (95% CI: 0.91, 1.90; p < 0.001) during the pandemic period compared to the pre-pandemic period (Table 3, Figures 2-3).

**Table 3:** Mean daily breathing problem-related EMS call volume and non-transport rate, expressed as percentages of total daily EMS calls, across the pre-pandemic, pandemic, and post-pandemic study periods Values are presented as a percentage of the total daily volume of EMS calls, with 95% CIs shown for each group. EMS: Emergency Medical Service, CI: confidence interval

Period	Mean daily EMS volume for breathing problems as a percentage of total daily EMS calls (95% CI)	Mean daily EMS volume of non-transports as a percentage of total daily EMS calls (95% CI)
Pre-pandemic	9.05% (8.89, 9.22)	15.61% (15.38, 15.83)
Pandemic	10.46% (9.99, 10.92)	19.90% (19.27, 20.53)
Post-pandemic	9.37% (9.25, 9.50)	17.60% (17.43, 17.77)

**Figure 4:**
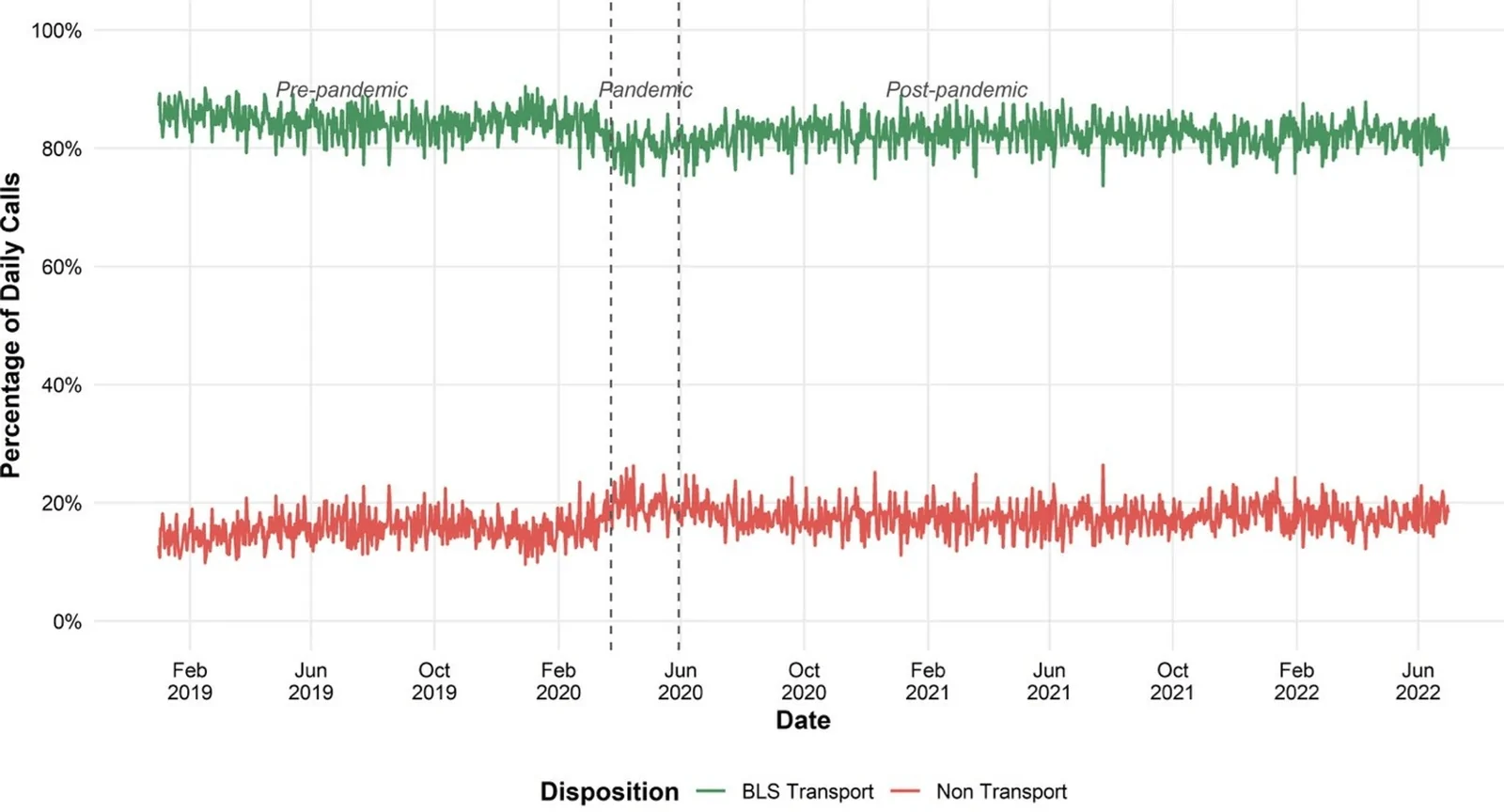
Average daily EMS utilization by percentage of call volume resulting in patient transports and non-transports during the pre-pandemic, pandemic, and post-pandemic periods EMS: Emergency Medical Service, BLS: basic life support

Total transports and percentage of activations were 371,379 (83%). Pre-pandemic transports totaled 131,581 (84.5%), pandemic transports totaled 15,681 (80.1%), and post-pandemic transports totaled 224,117 (82.4%). Non-transports, which include patient refusals, totaled 76,034 (17.0%) overall. Mean daily percentages of non-transports were 15.61% pre-pandemic (95% CI: 15.38%, 15.83%), 19.90% pandemic (95% CI: 19.27%, 20.53%), and 17.60% post-pandemic (95% CI: 17.43%, 17.77%). Compared to pre-pandemic, the occurrence of non-transports increased by 4.29 (95% CI: 3.62, 4.96) during the pandemic and 1.99 (95% CI: 1.71, 2.28) post-pandemic, with both being statistically significant (p < 0.001) (Tables 3-4, Figure 4).

**Table 4:** Total volume of EMS transports and non-transports, along with their percentages of total EMS calls, across the pre-pandemic, pandemic, and post-pandemic study periods EMS: Emergency Medical Service

Category	Transports (n, %)	Non-transports (n, %)
Pre-pandemic	131,581 (84.5%)	24,200 (15.5%)
Pandemic	15,681 (80.1%)	3,903 (19.9%)
Post-pandemic	224,117 (82.4%)	47,931 (17.6%)
Overall	371,379 (83.0%)	76,034 (17.0%)

## Discussion

Overall EMS utilization during the COVID-19 pandemic decreased significantly, and calls resulting in non-transports increased significantly during the pandemic period in Delaware. This study builds on existing literature showing decreased EMS calls and increased refusals for transport during the pandemic [1-4,6]. "Fear of seeking care in EDs and hospitals treating COVID-19-positive patients is suspected to have contributed to the decrease in volume. Fewer calls for non-respiratory complaints, with a concurrent increase in cardiac arrests, support avoidance of care for non-COVID-19 complaints during the pandemic [3,6,10]. Public perception of danger, governmental lockdowns, and changes in lifestyle patterns are hypothesized to have played a role in these effects. Further analysis is needed to better understand EMS utilization during pandemic months.

Percentages of calls for respiratory complaints increased in Delaware during the pandemic. This data is consistent with the existing literature [2,3,5]. The increase in respiratory concerns may represent any combination of the COVID-19 disease process, limited access to care, or public fear of the illness despite fewer overall EMS activations during the pandemic.

While not significant, the data trended toward an overall increase in EMS volume in the two years following the initial pandemic phase in Delaware. When compared to Strum et al., which found a significant increase in post-pandemic call volume, the difference in time period selected for the pandemic is notably longer than that selected in this study [8]. The behavioral changes in healthcare utilization that resulted from COVID-19 likely persisted beyond the cessation of stay-at-home orders. Further research is needed to understand long-term change in healthcare utilization.

To our knowledge, this is the first study in the US examining the post-pandemic impact of COVID-19 on EMS utilization. The increased volume is particularly concerning in the setting of a strained post-pandemic healthcare system. A study from Iran on post-pandemic EMS volume from 2020 to 2023 noted an increase in accidents (encompassing all forms of traffic accidents) in a seasonal distribution [11]. The increase in volume may represent a rebound following COVID-19 lockdowns. A post-COVID-19 reduction in the healthcare workforce [12,13] may also have contributed to an increased burden on EMS. Alternatively, this phenomenon may be consistent with an expected increase based on population.

Further research on post-pandemic EMS volumes and utilization patterns is needed to fully understand the long-term impact of the COVID-19 pandemic on healthcare and EMS utilization. A breakdown of EMS utilization in the first two years of the pandemic, with the release of a vaccine and return of restrictions during subsequent variants, may help elucidate patterns. Understanding changes in utilization during and after a pandemic is important for informing future prehospital resource allocation.

Limitations

The findings from this study are subject to limitations of any retrospective review. Some values such as race and ethnicity were missing for nearly half of the cases. The study only included the documented demographic data as reported in the PCRs, and there could have been a more substantial demographic shift during the compared time periods that was not reflected in the data as recorded. The 2020 Decennial Census was utilized for population data at the time of this study, which may not accurately reflect any population changes that occurred during the study periods.

Delaware’s statewide EMS structure includes a common dispatch model, a single statewide electronic PCR system, a two-tiered BLS/ALS response, and a relatively small geographic footprint compared to other US states. The retrospective data presented in this study represent general EMS patterns throughout the entire state across urban, suburban, and rural environments. They may generalize to more diverse EMS systems throughout the US. More localized or homogeneous EMS systems may have experienced different EMS utilization patterns during the same periods that were included in this study.

The study also examined three time periods of varying lengths; however, this was controlled by comparing the daily averages and percentage rates of daily call averages within the time periods. The decision to use the Delaware stay-at-home order to define the pandemic period may not fully capture pandemic behavior changes that occurred prior to COVID-19 vaccination or during the second and third waves of COVID-19 variants that occurred within 18 months of the initial variant.

Only the respiratory complaint as noted at the time of dispatch was assessed, which may be subjective due to potential dispatcher bias. Alternatively, although data were available regarding EMS provider impressions of primary patient complaints, a large variety of respiratory-related options were available, which may introduce another level of provider bias. Respiratory complaint by dispatch was determined to be more easily comparable between time periods.

Finally, patient non-transports included patient refusal, no patient found, public assist, or canceled. While the research team assumed that the patient refusal to transport accounted for the majority of observed differences in the patient non-transport category, there may have been additional factors that contributed to these values during the study periods.

Despite the limitations, this study provides important insight into EMS utilization before, during, and after a global pandemic. It carries implications for future research and EMS resource planning for future health crises.

## Conclusions

Significant shifts in statewide EMS utilization occurred during the COVID-19 pandemic in Delaware. EMS activations for respiratory complaints and rates of patient non-transports increased in Delaware during the pandemic. Transient decreases in EMS call volume during the pandemic stay-at-home orders have rebounded to pre-pandemic levels in Delaware. Statewide data in Delaware demonstrated a trend toward a post-pandemic increase in EMS volume. An increase in post-pandemic EMS utilization would have broad implications for staffing and future prehospital and hospital utilization planning. These findings regarding EMS call volume and transport volume can help inform planning for future pandemic events within the Delaware EMS system.
